# Diagnostic imaging in bronchial atresia

**DOI:** 10.1590/0100-3984.2021.54.2e1

**Published:** 2021

**Authors:** Alessandro Severo Alves de Melo

**Affiliations:** 1 Universidade Federal Fluminense (UFF), Niterói, RJ, Brazil. Email: alesevero@gmail.com.

Bronchial atresia is a rare congenital anomaly that is characterized by the obliteration of a lobar, segmental, or subsegmental bronchus. It is of unknown pathogenesis and is usually asymptomatic, typically being diagnosed on the basis of an incidental finding in adulthood. In most cases, it affects the left upper lobe. The lung tissue distal to the obliterated bronchial segment may show normal development, due to the arrival of air through collateral routes, resulting in localized hyperinflation and air trapping^(1-4)^.

The chest X-ray findings of bronchial atresia are generally nonspecific, although, upon careful observation, they can raise suspicion of the diagnosis. The X-ray finding most suggestive of bronchial atresia is an elongated opacity accompanied by a focus of hyperinflation. A chest X-ray obtained during expiration can show air trapping. Therefore, computed tomography (CT) of the chest should be performed in patients suspected of having bronchial atresia^(4,5)^.

Chest CT is the gold-standard method for the diagnosis of bronchopulmonary diseases and is crucial for establishing the diagnosis of bronchial atresia. The main CT findings of bronchial atresia are mucocele and an area of hyperinflation distal to the affected bronchial segment, together with air trapping. A loss of continuity in the affected segment, especially discontinuity of the mucocele in relation to the central bronchi, can also be seen^(1)^. In the presence of a mucocele, it is essential to exclude the diagnosis of endobronchial lesions, such as bronchogenic carcinoma, a foreign body, and a broncholith. In most cases, that distinction can easily be made by chest CT. In an imaging context consistent with bronchial atresia, a finding of centrilobular nodules, nodules in the airspace, or areas of consolidation is likely related to a concomitant infectious process. Bronchial atresia may be accompanied by other congenital anomalies, such as congenital pulmonary airway malformation, lobar hyperinflation, and pulmonary sequestration^(1-5)^.

The article authored by Di Puglia et al.^(6)^, published in the previous issue of **Radiologia Brasileira**, addresses, in an in-depth and substantiated manner, a significant number of cases of bronchial atresia, evaluating the CT findings and confirming multiple aspects described in the literature^(1-4)^. Their article is relevant mainly because it sheds light on a subject that has been little studied and is therefore not very well known to physicians, especially radiologists. In their study sample, patient age at diagnosis varied widely, which is attributable to the fact that most patients with bronchial atresia are asymptomatic, resulting in late diagnosis in the majority of cases. In the pediatric age group, the diagnosis is typically made during the imaging investigation of recurrent infections or during imaging examinations performed for other reasons, such as preoperative evaluation^(7-9)^. Another reason for the late diagnosis of bronchial atresia is the relative ignorance of the condition on the part of physicians, which makes the Di Puglia et al.^(6)^ study even more relevant.

Another notable aspect of the Di Puglia et al.^(6)^ study is the detailed description of CT patterns. In most cases, mucoceles exhibit a branched pattern, and some have a rounded morphology. Most are fluid-filled, although some are air-filled. In general, bronchial atresia results in unilateral pulmonary involvement, typically in the left upper lobe or right lower lobe^(1-4)^. Di Puglia et al.^(6)^ underscore the concept that mucocele, distal hyperinflation, and hypovascularization (hypovolemia), which were observed in 100% of their cases, are characteristic of bronchial atresia, as has previously been reported in the literature^(1-4)^.

In conclusion, by studying the CT patterns of bronchial atresia in detail and in a significant number of cases, Di Puglia et al.^(6)^ have made a major contribution to the diagnosis of the condition by imaging.
